# Book Notices

**Published:** 1889-04

**Authors:** 


HAND BOOK OF HISTORICAL AND GEOGRAPHICAL PHTHISISOLOGY, with special reference to the distribution of consumption in the United States. Compiled and arranged by Geo. A. Evans, M. D., N. Y. pp. 295. D. Appleton & Co., 1888.

This is a valuable little book for all that are interested in health resorts for consumptives. It gives the geographical distribution of consumption in the United States and the death rate in every climate. Texas is mentioned as one ot the resorts in the United States, that gives the greatest immunity from consumption. Bœrne, Kendall county, is especially noticed. The author gave three pages to Texas, and had he been more familiar with the climate, he would have given a dozen, for Texas possesses the greatest variety of climate between extremes of any country in the world; and variety of climate for health is just as necssary as variety of food.

---

WOODS' MEDICAL AND SURGICAL MONOGRAPHS, Vol. 1, No. 1 for January, 1889. Contents: "The Pedigree of Disease," by Jonathan Hutchinson, F. R. S. "Common Diseases of the Skin," by Robert M. Simon, M. D. "Varieties and Treatment of Bronchitis," by Dr. Ferrand.

Vol. 1, No. 2. Contents: "Gonorrhœal Infection in Women," by William Japp Sinclair, M. A., M. D. "On Giddiness," by Thomas Grainger Stewart, M. D. "Albuminuria in Bright's Disease," by Dr. Pierre Jaeriton.

These books are published monthly by William Wood & Co., New York. They contain about 250 pages each. Price $10.00 a year, single copies, $1.00.

This enterprising publishing company, having secured the best medical writers to be found in any country, are furnishing literature of the highest practical and scientific value for less money than ever before. The two numbers at hand, each containing six articles, would be cheap at $2.00 apiece.

